# Eclampsia

**Published:** 1910-04

**Authors:** O. L. Stephenson

**Affiliations:** Roanoke, Ala.


### ECLAMPSIA.

O. L. STEPHENSON, M. D., ROANOKE, ALA.

When your president approached me in regard to writing a paper for this occasion, we were just concluding an 18 hour siege with that dreadful disease Eclampsia. He suggested that I take Eclampsia as my subject. I cannot hope to offer you anything new or startling in the handling of this malady, but if I shall be able to impress upon your minds some of the old facts which you may have forgotten, I shall have accomplished all that I hope for. I have been unfortunate in having to deal with this condition in no less than six cases during the 6 years of my professional life, occurring in my own practice, and in those of my conferees where I have been called in consultation. Of these 6 cases there was one maternal and two foetal deaths. One of the fatal cases was in a patient who was about seven months advanced in pregnancy, and in whom we were unable to effect delivery.

Statistics go to show that Escampsia occurs in 1 to 500 labors, but my experience is, and that of other physicians with whom I have discussed this subject, leads me to believe that in the small towns and country the disease is much more frequent. This I believe to be due largely to the fact that many of our patients do not come under the physician's observation till labor actually begins, or the patient is seized with convulsions.

About 75 per cent. of the cases occur in primiperae. In my cases all were primiperae. Twin pregnancy and hydramnios are said to be predisposing factors and some authorities claim that heredity plays an important role in the causation. In my cases two occurred in the same family.

Pathology: Formerly it was believed that Eclampsia was altogether due to a failure of elimination by the kidneys and was always accompanied by albuminuria or a greatly decreased output of urea. Later researches have proven that Eclampsia often occurs without the urine showing any abnormality on chemical analysis, and the doctor who contents himself with a cursory urinalysis and fails to see that the other emunctories are properly at work, is due and will eventually receive a rude awakening from his fancied security. Allow me at this point to call your attention to the especial importance of seeing that the liver is acting properly. For I beg to assure you that it is frequently more at fault than the kidneys. Indeed, the last case I attended I feel sure the Eclampsia was the result of failure hepatic elimination. I was somewhat suspicious of her as her sister had suffered an attack about two years prior, and I kept a vigilant watch on her kidneys and bowels, and was getting good elimination from both sources, and two urinalysis made a few days prior to the beginning of labor showed no abnormality either in the quality or the quantity of the urine. The patient was a pronounced brunette, and of a family subject to liver troubles, all being of the so-called bilious temperament. A few minutes after the first convulsion she began vomiting large quantities of green material, and when purgation was established many copious stools of the same character were obtained. I am confident that if I had administered calomel to her in generous doses about twice a week for a few weeks prior to confinement I would have prevented the attack. She had no headache, but slight edema of lower limbs, no disturbance of vision, or other prodromal symptoms which usually accompany kidney insufficiency.

A condition know as Hemorrhagic Hepatitis was demonstrated in 1886 by Jurgens and Klebs to exist in the livers of all the cases which they examined of patients dying of Eclampsia. These hepatic changes have in more recent years been found to be practically always present. In many instances they are easily demonstrated macroscopically. We cannot escape the conclusion therefore that failure of hepatic function has largely to do with the condition.

In from 75 to 80 per cent. of autopsies various brain lesions have been found, consisting of edema, congestion, thrombosis, or hemorrhage.

Williams says, it is apparent therefore that the main lesions in Eclampsia are found in the kidneys, liver and brain; those in the liver being more constant. Accordingly, we are forced to conclude that under the term Eclampsia are included a number of different disease entities, each with their own anatomical lesions, or what is more probable, that the morbid condition is caused by some as yet unknown poisonous substance circulating in the blood which may give rise to lesions of varying intensity in the several organs.

From the foregoing it is evident that the etiology of the disease is still in a case of obscurity, and I shall not trespass upon your time to even enumerate the various theories that have been advanced as to its origin. Zweifel has described it as the "disease of theories."

*Diagnosis*:—The diagnosis is usually apparent, and has only to be differentiated from hysteria, Epilepsy, and Uremia, which considering the history is usually quite easy.

*Prognis*:—This should always be quite guarded as the condition is very grave and the patients often die suddenly after only one of two convulsions from Apoplexy, heart failure or other complication. The average of statistics I have consulted places the maternal mortality at about 25 per cent. and the foetal at 50 per cent.

*Treatment*:—This naturally divides itself into Prophylactic and Curative, of which the former is the more important if not so spectacular, and is certainly much safer for the patient. It is quite certain that if our patients would place themselves under our care earlier in pregnancy and if we would keep a more watchful surveilance over the processes of metabolism our cases would be much fewer. We should see that the elimination of urea is up to the standard, and that sufficient urine is voided and what is of equal if not more importance, that the liver and bowels are acting freely. And let me here again emphasize the extreme imI portance of liver excretion, for in many cases I believe the fault lies there and not in the kidneys. Allow me to say just here that authorities are agreed that cases in which edema is present are more amenable to treatment and less likely to have Eclampsia than those in which it is absent, hence our fears of Eclampsia on account of edema are not so well founded as we formerly supposed.

*Curative*:—During the attack Chloroform by inhalation to control the convulsions, at the same time protecting the patient from biting her tongue and lips by inserting between her teeth a piece of wood or the handle of a spoon wrapped with a piece of cloth. Hypodermics of morphine to pronounced narcosis are highly praised by most authorities, though a few condemn them as tending to lock up secretions. In that form of Eclampsia where the coma is pronounced and the patient totally unconscious, I do not believe that morphine is indicated, but in the cases where the patient is actively delirious, showing great cerebral excitement I think it not only indicated, but imperatively demanded. I confess that I have never made this distinction in actual practice as I am always too anxious to give everything that I have ever heard recommended for the condition. It is supposed to act by benumbing the brain centers and rendering them less sensitive to the irritation of the circulating poison in the blood. Chloral and the Bromides, which must usually be given per rectum act in much the same way, and also produce an anemia of the brain, thus counteracting the tendency to cerebral hemorrhage which is almost if not quite constantly present. Perhaps next in order of importance, as regards drug medication is Ver. Vir.

It is somewhat remarkable that such a diversity of opinion should exist in regard to the efficiency of this drug. Many physicians consider it the remedy sine qua non, while others either ignore it entirely or else give it a very insignificant place on the list.

I believe much of this diversity is due to the fact that a proper and discriminating diagnosis is not made between the various types of the disease. In the cases where there is a full, bounding and rapid pulse I believe that Ver. given in liberal doses and given to effect, i. e., till the pulse slows to 60 or 70 per minute and assumes a soft compressible feeling to the touch, will do a work that no other drug will accomplish. I have learned from a sad experience, however, that there are some cases in which it is impossible to produce the physiological effect of the drug, even when given in doses aggregating about 140 gtt. in the course of about 3 hours. In cases where the temperature is high, the skin hot and dry, and the pulse quite full, I believe good would accrue from full doses of Phenacetine or Aceanilid, if the patient can be induced to swallow. I have never seen these drugs recommended, and I confess I have never tried them, but with symptoms as above described we might confidently use them for the following reasons: First, they would reduce the temperature more rapidly and more effectual than any other remedy: Second: they would soften the arterial pressure and equalize the circulation: Third, they would produce an anemia of the brain, thus relieving to a great extent the intense headache, which is so often a distressing and disturbing factor, and also by relieving the cerebral congestion tend to lessen the danger of cerebral hemorrhage: And last, but not least, they would induce a free diaphoresis, which we know is so much desired in these cases.
cerebral congestion tend to lessen the danger of cerebral hemmorrhage: And last, but not least, they would induce a free diaphoresis, which we know is so much to be desired in these cases.

Catharsis should be obtained as speedily and as copiously as possible. Croton Oil 1m to 2m to a teaspoonful of Olive Oil or Castor Oil is most effectual and easily administered. The mixture of the oils will not strangle the patient like an aqueous solution, and by placing far back on the tongue will usually be swallowed without difficulty. This should be followed as soon as practical with large doses of Calomel given dry on the tongue. Diaphoresis and diuresis should be stimulated by hot packs, and by hot saline enemas thrown high in the bowels, which owing to the unconscious condition of the patient will usually be retained in remarkably large quantities.

*Bleeding.*—If there is one condition above all others in which this once much misused, and now almost unused, means is indicated it is the sthenic type of Eclampsia. It is rational from the fact that it decreases the amount of poison in the circulation, and also relieves vascular tension, thereby rendering less likely cerebral or hepatic hemorrhage, which are such frequent and usually fatal accompaniments of this condition.

In my opinion and it is sustained by my experience and that of many others, the most important measure it to effect delivery as speedily as possible. And I use the expression as possible with a wholesome respect for the fact that this advice is sometimes more easily given than followed, for with two other doctors I once worked for several hours in an attempt to deliver a patient, and without success. And in another case with a competent physician I worked all night before delivery was effected. And in yet another case with an experienced colleague it required about 36 hours to accomplish delivery. In the majority of cases, however, labor is hastened if already begun, and if not actually in progress it is generally instituted soon after the convulsions have begun. The most effectual method of hastening labor is by forcible but careful dilation of the cervix. And the best instrument I have ever used is the hand. After dilation is sufficiently advanced an assistant to aid in putting the cervix on the stretch is of invaluable aid. As soon as dilation is far enough advanced apply forceps and deliver. Fortunately, this usually, though not always, ends the trouble. For while some cases continue to have convulsions after delivery, this is not the rule. Where such is the case, all that remains to be done is to keep the patient under the influence of the drugs before enumerated, and maintain free elimination, meeting any other complications as they arise. The most common of these complications are, Pulmonary Edema, heart failure, aspiration Pneumonia, and Apoplexy.

